# Delusional Distress is Associated With Emotion Dysregulation in Schizophrenia-Spectrum Disorders

**DOI:** 10.1093/schizbullopen/sgaf010

**Published:** 2025-05-23

**Authors:** Essence Leslie, Lauren P Weittenhiller, Ali F Sloan, Julia M Sheffield

**Affiliations:** Department of Psychiatry and Behavioral Sciences, Vanderbilt University Medical Center, 1601 23rd Ave S, Suite 3057K, Nashville, TN 37212, USA; Veterans Affairs Greater Los Angeles Healthcare System, 11301 Wilshire Blvd. Bldg. 206, Room 15, Los Angeles, CA 90073, USA; Department of Psychological Sciences, College of Arts and Science, Vanderbilt University, 2301 Vanderbilt Place, Nashville, TN 37240, USA; Department of Psychiatry and Behavioral Sciences, Vanderbilt University Medical Center, 1601 23rd Ave S, Suite 3057K, Nashville, TN 37212, USA

**Keywords:** emotion regulation, psychosis, self-efficacy, paranoia, strategies, affect

## Abstract

**Background and Hypothesis:**

The distressing nature of delusional beliefs is considered key to their persistence. One potential mechanism underlying delusional distress is global emotion dysregulation. Global emotion dysregulation is associated with general psychotic symptom severity, but its specific relationship to delusional distress has yet to be closely examined.

**Study Design:**

People with (*n* = 100) and without (*n* = 52) schizophrenia-spectrum disorders completed measures of delusional ideation (Peters Delusion Inventory; PDI-21) and global emotion dysregulation (Difficulties in Emotion Regulation Scale; DERS-16). Participants with schizophrenia also completed a measure of delusion severity (Psychotic Symptom Rating Scales; PSYRATS). Relationships between delusion severity and global emotion dysregulation were assessed with linear regression, controlling for age, sex, and group. Associations between delusional distress and aspects of emotion dysregulation were determined using stepwise linear regression.

**Study Results:**

Global emotion dysregulation was significantly elevated in those with schizophrenia compared to non-clinical controls, across all subscales (*P* < .001). Emotion dysregulation was significantly associated with delusional distress on both the PDI-21 (*P* < .001) and PSYRATS (*P* < .001). Stepwise regressions revealed a specific association between limited access to emotion regulation strategies and delusional distress on both scales (*P*’s < .001). Delusional distress remained associated with emotion dysregulation when controlling for delusional preoccupation, and emotion dysregulation was not significantly associated with delusional conviction on either scale.

**Conclusions:**

Emotion dysregulation, particularly the sense that there is little one can do to regulate themselves when upset, relates to delusional distress. Interventions that increase access to more emotion regulation strategies may help decrease distress associated with delusional thinking.

## Introduction

Delusions are a hallmark symptom of schizophrenia-spectrum disorders, defined as fixed beliefs that are not amenable to change in light of conflicting evidence (DSM-V-TR). Delusions exist on a continuum of severity, with more severe forms relating to lower levels of well-being, increased suicidal ideation, and poorer recovery from daily life stressors.^1–3^ Delusion severity consists of multiple dimensions, typically defined as: (1) conviction, the degree to which an individual believes that the delusional belief is true, (2) preoccupation, the amount of time and the extent to which the delusion occupies someone’s mind, and (3) distress, how distressing (ie, emotionally upsetting) an individual finds the delusion to be.^4^ While delusional conviction is often the focus of clinical interventions,^5,6^ delusional distress is increasingly recognized as impacting the persistence of delusional thinking, contributing to progression and maintenance of psychosis.^7–9^

Understanding the mechanisms that contribute to delusional distress is imperative for improving clinical interventions. Given the nature of distress, as a subjectively negative affective state, emotional processes may be a particularly useful target. This is true across the psychosis spectrum. Findings from the Adolescent Brain Cognitive Development study found that adolescents who experience distressing delusional ideation have higher instances of psychopathology, greater mental health service utilization, and more impaired cognition and functioning compared to people reporting low levels of distress related to their delusional ideation.^10^ In subclinical populations, people who experience distressing hallucinations are 4 times more likely to develop delusions compared to people who experience hallucinations that were not distressing.^7^ In clinical populations, delusional distress is strongly associated with worry and meta-worry, and reducing worry severity significantly improves delusional distress.^8,9^ Together, these data suggest that distress associated with psychotic experiences, as opposed to simply the presence or absence of the experience itself, is an important component of its severity and persistence.

One potential contributor to delusional distress is global emotion dysregulation. Global emotion dysregulation refers to a broad deficit in one’s understanding of and relationship to emotions, without assessing the use of specific cognitive or behavioral strategies.^11^ While impairments in cognitive emotion regulation strategies have been documented in schizophrenia,^12^ less is known about how self-assessed global emotion dysregulation difficulties may influence delusion severity. Gratz and Roemer’s model of global emotion dysregulation includes 4 distinct components: (1) awareness, understanding, and acceptance of emotions; (2) the ability to engage in goal-directed behaviors and inhibit impulsive behaviors when experiencing negative emotions; (3) the ability to use situationally appropriate strategies to modulate emotional response; and (4) the willingness to experience negative emotions as a part of meaningful life experiences.^13,14^ The brief version of the Difficulties in Emotion Regulation Scale (DERS-16) is a validated measure that assesses these components of global emotion dysregulation, with the exception of emotional awareness. For component (1), the DERS-16 captures lack of emotional clarity, reflecting difficulties in identifying and understanding one’s emotions. For component (2), it assesses difficulties engaging in goal-directed behavior (ie, challenges concentrating and accomplishing tasks when experiencing negative emotions) and impulse control difficulties (ie, problems controlling behavior when experiencing negative emotions). Component (3) is measured by limited access to emotion regulation strategies—the belief that little can be done to manage emotions when upset. Component (4) is measured by nonacceptance of emotional responses, reflecting negative secondary reactions to one’s own negative emotions. In subclinical populations, distress associated with psychotic-like experiences is related to global emotion dysregulation,^15^ and distress associated with paranoia is related to greater nonacceptance of emotional responses.^16^ Meta-analyses have found that both global emotion dysregulation and the use of maladaptive cognitive emotion regulation strategies are increased in schizophrenia and associated with positive symptoms.^12,14,17^ The relationship between global emotion dysregulation and delusional distress in schizophrenia-spectrum disorders has not been specifically examined.

The current study aims to further our understanding of contributory factors to delusional distress by examining relationships with self-reported global emotion dysregulation. In a relatively large sample of well-characterized individuals with schizophrenia-spectrum disorders, we aim to: (1) report differences in global emotion dysregulation and its dimensions between patients with schizophrenia-spectrum disorders and healthy comparison participants; (2) examine differences in delusional ideation and its factors (conviction, preoccupation, distress) between patients with schizophrenia-spectrum disorders and healthy comparison participants; and (3) assess whether global emotion dysregulation and/or any of its specific dimensions are associated with distress of delusional ideation and/or clinical delusions. We also conducted sensitivity analyses controlling for preoccupation and negative affect.

## Methods

A total of 100 participants with a schizophrenia-spectrum disorder (53 people with schizophrenia, 28 with schizoaffective disorder, 10 with schizophreniform, 6 with delusional disorder, and 3 with brief psychotic disorder) and 52 non-clinical comparison participants were recruited across 2 different studies (IRB #202462 and IRB #201489) (Table 1). Participants with schizophrenia/schizoaffective disorder did not differ from those with other schizophrenia-spectrum disorder diagnoses on any demographic to symptom severity variable (see Table S1). Participants with psychosis were between 18 and 65 years old and were identified from the Vanderbilt Psychiatric Hospital and from the Vanderbilt University Medical Center Psychotic Disorders Program. Diagnosis was determined by the patient’s psychiatrist and confirmed by medical records review and/or completion of modules A and B of the Structured Clinical Interview of the DSM-5 or DSM-IV (SCID),^18^ conducted by a trained psychologist. The majority of participants with schizophrenia-spectrum disorders were taking at least one anti-psychotic medication (*n* = 95; 95%), 55% of participants had participated in psychotherapy within the last month (*n* = 55), and 52% of participants were either unemployed or receiving government assistance (*n* = 52). The non-clinical comparison group were between the ages of 18–55 years and were asked if they had any first-degree relatives that had a psychotic disorder diagnosis. In addition, a Structured Clinical Interview of the DSM-IV or 5 was collected on all non-clinical participants and they did not meet criteria for schizophrenia-spectrum personality disorders. Non-clinical comparison participants were allowed to meet criteria for a past mild depressive episode or past mild substance use disorder but did not meet criteria for any current mental health disorders and could not be currently prescribed psychotropic medication. Across both groups, history of a neurological illness (eg, epilepsy) or traumatic brain injury resulting in loss of consciousness of over 30 min were exclusion criteria. Premorbid intelligence scores were determined by the Weschler Test of Adult Reading, and potential participants that had standard scores of < 79 were excluded from the study.^19^

**Table 1.** Participant Demographics

Variable	Controls *N* = 52^1^	SSD *N* = 100^1^	Test statistic and *P*-value
Age, y	29.3 (7.0)	29.9 (9.6)	*t*(133.7) = −0.41, *P* = .682
Gender			χ² = 0.71, *P* = .91
Cisgender Man	34 (65%)	68 (68%)	–
Cisgender Woman	17 (33%)	29 (29%)	–
Nonbinary/Genderfluid/Gender queer	1 (1.9%)	2 (2.0%)	–
Transgender (FTM)	0 (0%)	1 (1.0%)	–
Ethnicity			χ² = 0.01, *P* = 1
Hispanic	5 (9.6%)	10 (10%)	–
Not Hispanic	47 (90%)	90 (90%)	–
Race			χ² = 10.99, *P* = .0775
American Indian or Alaskan Native	0 (0%)	2 (2.0%)	–
Asian	3 (5.8%)	2 (2.0%)	–
Black or African American	12 (23%)	39 (39%)	–
Middle Eastern/Northern African	1 (1.9%)	1 (1.0%)	–
Multiracial or more than 1 race	0 (0%)	5 (5.0%)	–
Other	1 (1.9%)	4 (4.0%)	–
White	35 (67%)	47 (47%)	–
Personal education, y	17.0 (2.3)	13.7 (2.4)	*t*(109) = 8.14, *P* ≤ .001
Parental education, y	15.5 (2.7)	14.8 (2.6)	*t*(108.1) = 1.51, *P* = .135
PDI-21 total scores	14.5 (21.4)	79.8 (59.6)	*t*(66.9) = −7.55, *P* ≤ .001
PDI-21 distress	0.8 (1.0)	2.2 (1.2)	*t*(101.7) = −6.51, *P* ≤ .001
PDI-21 preoccupation	0.9 (1.1)	2.3 (1.2)	*t*(103.5) = −6.68, *P* ≤ .001
PDI-21 conviction	1.5 (1.8)	2.9 (1.2)	*t*(89.3) = −4.74, *P* ≤ .001
DERS-16 total scores	22.9 (6.9)	40.1 (16.8)	*t*(144.2) = −8.92, *P* ≤ .001
Goals	1.8 (0.8)	2.9 (1.3)	*t*(144.4) = −6.67, *P* ≤ .001
Nonacceptance	1.6 (0.7)	2.5 (1.2)	*t*(149.2) = −6.11, *P* ≤ .001
Strategies	1.3 (0.5)	2.5 (1.1)	*t*(146) = −8.88, *P*≤ .001
Impulse	1.1 (0.3)	2.1 (1.1)	*t*(117.3) = −8.69, *P* ≤ .001
Clarity	1.4(0.6)	2.4 (1.3)	*t*(149.9) = −6.21, *P* ≤ .001
PSYRATS total	–	12.8 (6.1)	–
PSYRATS distress	–	3.9 (3.03)	–
PSYRATS preoccupation	–	4.6 (2.9)	–
PSYRATS conviction	–	2.9 (1.1)	–

^1^Mean (SD); *n* (%); SSD = schizophrenia-spectrum disorders; y = years.

## Study Measures

### Global Emotion Dysregulation

Global emotion dysregulation was assessed using the Difficulties in Emotion Regulation Scale (DERS-16). The DERS-16 is a widely used self-report measure designed to assess difficulties in emotion regulation across various dimensions.^20^ Participants respond to each item on a 5-point Likert scale, ranging from 1 (almost never) to 5 (almost always). Higher scores indicate greater difficulties with emotion regulation. While the original DERS-36 consists of 6 subscales of global emotion dysregulation, The DERS-16 consists of 5 of the 6 subscales, omitting the sixth subscale of poor emotional awareness. NonAcceptance of Emotional Response is a subscale measure of a person’s tendency to have negative secondary reactions to their emotional experiences, such as feelings of shame, guilt, or frustration in response to emotions. (ie, “When I’m upset, I feel guilty for feeling that way”). Difficulties Engaging in Goal-Directed Behavior evaluates how emotions interfere with a person’s ability to focus and complete tasks. Higher scores suggest that the person struggles to maintain focus and stay productive when experiencing negative emotions (ie, “When I’m upset, I have difficulty getting work done.”). Impulse Control Difficulties measures a person’s difficulty controlling behaviors and impulses when emotionally upset (ie, “When I’m upset, I feel out of control.”). Limited Access to Emotion Regulation Strategies evaluates the perceived effectiveness of emotion regulation strategies available to a person. High scores indicate a sense of helplessness, or that the person believes there are few ways to alleviate negative emotions effectively (ie, “When I’m upset, I believe that there is nothing I can do to make myself feel better.”) Lack of Emotional Clarity measures how well a person understands and identifies their emotions. High scores suggest difficulty in distinguishing between different emotions or understanding what one is feeling. (ie, “I have difficulty making sense of my feelings.”).

Each subscale score is calculated by summing the responses of the questions corresponding to that subscale, with higher scores reflecting greater emotion regulation difficulties. The overall DERS-16 score is the sum of all item responses, providing a global measure of emotion dysregulation.

### Delusional Ideation

Given prior work showing associations between emotion regulation and delusional ideation in subclinical populations,^15^ we wanted to examine whether global emotion dysregulation relates to distress of delusional ideation across our entire sample, in line with the view of a psychosis continuum.^21^ The Peters Delusion Inventory—21 Item Version (PDI-21) is a self-report measure designed to assess delusional ideation in both clinical and non-clinical populations.^22^ The PDI-21 is a shortened version of the original 40-item PDI, and it retains the same core structure, with a focus on the severity and impact of delusional thoughts. Each of the 21 items in the PDI-21 assesses the presence of a specific delusional idea, such as paranoia, grandiosity, or magical thinking. For each belief, participants are asked to rate their experience along 3 dimensions: distress, preoccupation, and conviction. Responses are provided on a 5-point Likert scale, ranging from 0 (not at all) to 4 (very much). Higher scores indicate greater severity of delusional ideation. Each of the 21 items is first scored as 0 (no delusional belief present) or 1 (delusional belief present). For each endorsed delusional belief, participants then rate it on the 3 subscales: distress, preoccupation, and conviction. The total score for the PDI-21 is the sum of all responses, with higher scores indicating greater delusional ideation. When examining specific subscales, we calculated “relative” scores, which took into account the number of items endorsed. For instance, the summed distress scores were divided by the number of items endorsed on the scale, so as not to overweight participants who endorsed more items.

### Clinical Delusions

The Psychotic Symptom Rating Scales (PSYRATS—Delusions Scale) is a clinician-administered interview tool designed to assess the severity of delusions across the dimensions of distress, preoccupation, conviction, and impact on functioning.^23^ With a clinically trained research assistant, participants developed a statement capturing one core delusion that encompassed most, if not all, of the current delusional thought content that was most prominent to the participant. These delusions are much more specific to the person’s day-to-day experience and clinical picture, compared to the more general ideations that participants can endorse on the PDI-21. Scores range from 0 to 24, with higher scores indicating more severe delusions. Participants were asked a total of 6 questions about this specific delusion, with 2 questions assessing distress, 2 questions assessing preoccupation, and one question each assessing conviction and impact on functioning.

### Data Analysis

All data were analyzed in R Studio (version 4.2.1). One-way ANCOVAs (analysis of covariance) were conducted to determine group differences between non-clinical controls and people with psychosis across all dimensions of the PDI-21 (distress, preoccupation, and conviction) and the DERS-16 (total scores and each of the 5 dimensions) controlling for age and sex. Linear regressions were used to test if global emotion dysregulation significantly predicted delusion severity across different domains, controlling for age, group (included as a dichotomized variable in all relevant analyses), and sex, with a group by emotion dysregulation interaction term. Backward stepwise linear regressions were used to investigate which specific aspects of global emotion dysregulation related to delusional distress on the PDI-21 and PSYRATS. In stepwise regression models, all DERS subscales were included as independent variables, along with age, sex and group (for PDI-21), predicting delusional distress. The Akaike Information Criterion (AIC) was used as the criterion for model selection. Starting with the full model, predictors were removed one at a time if their exclusion resulted in a lower AIC. Normality of all regression models in the manuscript were supported by Q-Q plots and non-significant Kolmogorov–Smirnov tests (all *P*’s > .05). Homoscedasticity was assessed via visual inspection of residuals and confirmed by non-significant Breusch-Pagan tests for all linear regression models (*P*’s > .05). Multicollinearity of stepwise regression models were tested using a variance inflation factor (VIF). All DERS subscales had VIF scores < 2.6 (with VIF = 1 indicating no multicollinearity, and a cutoff of 5 indicating concerns for multicollinearity), indicating some intercorrelation of DERS subscales but not enough to violate any model assumptions.

## Results

### Group Differences

**Figure 1.**
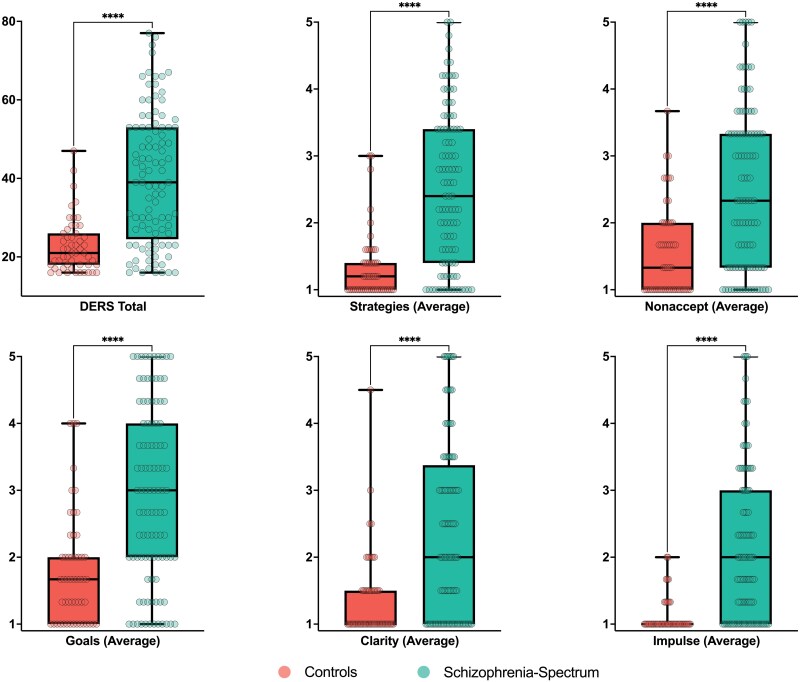
Schizophrenia-Spectrum Disorder Participants Demonstrated Significantly Reduced Emotion Regulation Abilities on the DERS-16, Including All Subscales

The schizophrenia-spectrum group reported greater overall global emotion dysregulation than the non-clinical control participants (*F*(1, 148) = 51.1, *P* < .001), which was significantly elevated across all subscales of the DERS-16 (all *P*’s < .001) (Figure 1). Delusional ideation was also significantly elevated in patients overall (*F*(1, 102) = 54.7, *P* < .001), and for each dimension: distress, preoccupation, and conviction (all *P*’s < .001). Intercorrelations between all variables included in the study, separated by group, are presented in the Supplementary Materials.

### Global Emotion Dysregulation and Total Delusion Severity

**Figure 2.**
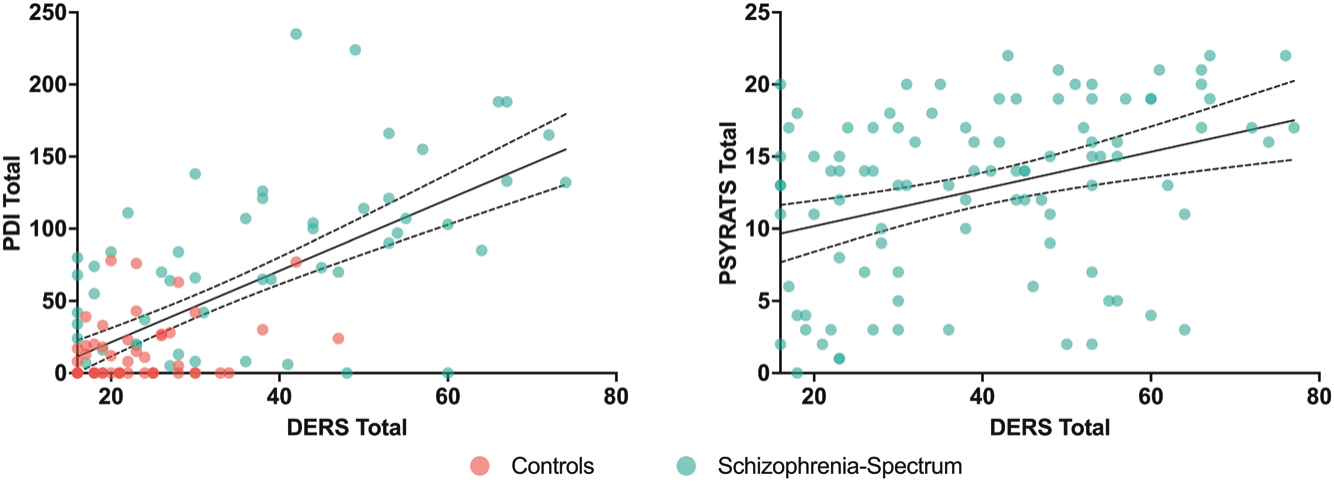
Global Emotion Dysregulation was Associated With Delusional Ideations and Clinical Delusions in Control Comparisons and Individuals With Schizophrenia-Spectrum Disorders

Global emotion dysregulation was significantly related to total delusional ideation severity for the PDI-21 when controlling for age, sex, and group (*R*^2^ = 0.51, *b* = 1.83, β = 0.5, *P* < .001). There was no significant group by global emotion dysregulation interaction (DERS-16 total scores * group), suggesting similar associations between global emotion dysregulation and delusional severity across all participants (β = 0.90, *P* = .30). Global emotion dysregulation was also significantly related to overall delusion severity for core delusional beliefs on the PSYRATS in the schizophrenia-spectrum disorders group (*b* = 0.21, β = 0.43, *P* < .001) (Figure 2). Age was also a significant predictor of PSYRATS total scores, suggesting that older age was associated with greater overall delusion severity for clinical delusions (*b* = 0.21, β = 0.25, *P* = .005).

### Global Emotion Dysregulation and Dimensions of Delusions

**Figure 3.**
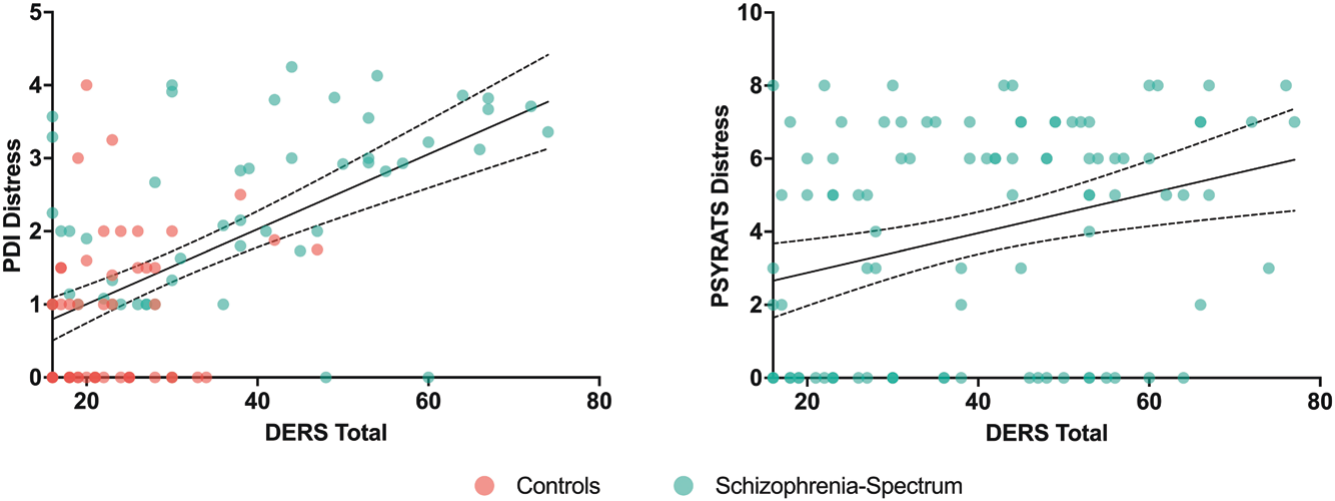
Global Emotion Dysregulation is Associated With Delusional Distress in Both Delusional Ideations and Clinical Delusions

Global emotion dysregulation was found to be strongly related to delusional distress on the PDI-21 (*R*^2^ = 0.40, *F*(6, 99) = 12.55, β = 0.41, *b* = 1.83, *P* < .001) and on the PSYRATS (*R*^2^ = 0.18, *F*(5, 93) = 5.18, β = 0.36, *b* = 0.21, *P* < .001), demonstrating that a substantial amount of delusional distress is associated with global emotion dysregulation (Figure 3). Age was also found to be strongly related to delusional distress on the PSYRATS (β = 0.32, *b* = 0.1, *P* = .0008), with older adults reporting greater distress. Global emotion dysregulation was also significantly associated with preoccupation rated on the PDI-21 (*b* = 0.05, β = 0.38, *P* = .004) and the PSYRATS (*b* = 0.06, β = 0.29, *P* < .001). Interestingly, global emotion dysregulation did not significantly relate to delusional conviction for the PDI-21 (β = 0.08, *b* = 0.009, *P* = .42) or the PSYRATS (β = 0.17, *b* = 0.01, *P* = .09).

To examine the specificity of the relationship between global emotion dysregulation and delusional distress, we investigated associations between global emotion dysregulation and distress, controlling for preoccupation, and vice versa. When controlling for preoccupation, global emotion dysregulation and distress were associated on the PDI-21 (β = 0.12, *b* = 0.005, *P* = .05) and the PSYRATS (β = 0.24, *b* = 0.03, *P* = .03); however, preoccupation was not significantly related to global emotion dysregulation when controlling for distress on the PDI-21 (β = 0.06, *b* = 0.005, *P* = .33) or PSYRATS (β = 0.13, *b* = 0.02, *P* = .15).

### Delusional Distress and Global Emotion Dysregulation Subscales

**Figure 4:**
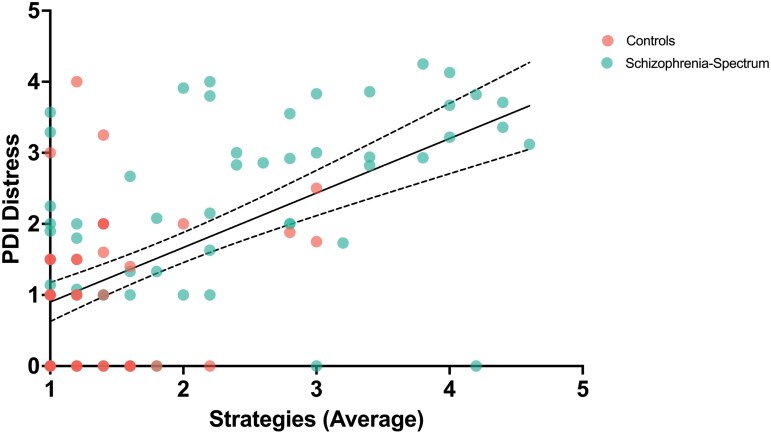
Limited Access to Emotion Regulation Strategies Predicted Delusion Severity on the PDI-21.

Backwards stepwise linear regression revealed that limited access to emotion regulation strategies, or feeling as though there is little one can do once they are upset, was the only global emotion dysregulation subscale that was significantly related to delusional distress (Figure 4). Critically, this was true for both the PDI-21 (*R*^2^ = 0.41, *F* (3,102) = 25.65, β = 0.32, *b* = 0.41, *P* < .005) and the PSYRATS (*R*^2^ = 0.26, *F*(3,95) = 12.24, β = 0.58, *b* = 1.53, *P* < .001). Interestingly, impulse control difficulties, or difficulties remaining in control of one’s behavior when experiencing negative emotions, were also related to elevated levels of delusional distress on the PSYRATS (*R*^2^ = 0.26, *F*(3,95) = 12.24, β = 0.31, *b* = −0.84, *P* < .006). Age continued to be a significant predictor of delusional distress for clinical delusions (β = 0.25, *b* = 0.08, *P* = .004). Sensitivity analyses were conducted to examine the impact of including measures of negative affect (depression and worry) in the model. We continued to find that emotion regulation self-efficacy (*P* = .001) and impulse control difficulties (*P* = .01) were the significant predictors of delusional distress, above and beyond negative affect (details are included in the Supplementary Materials).

## Discussion

This study examined the relationship between delusional distress and global emotion dysregulation in schizophrenia-spectrum disorders. We found that global emotion dysregulation was significantly related to the severity of both delusional ideation (across our whole sample) and clinical delusions of schizophrenia-spectrum disorders. Of the different delusion dimensions, we observed significant associations between global emotion dysregulation and delusional distress for both delusional ideation and clinical delusions. Critically, global emotion dysregulation remained associated with delusional distress on both scales when controlling for preoccupation; however, preoccupation was not significantly associated with global emotion dysregulation when controlling for distress. Interestingly, delusional conviction was not significantly related to global emotion dysregulation on either scale. For clinical delusions in our schizophrenia-spectrum disorder participants, older age was associated with greater overall delusion severity, greater global emotion dysregulation difficulties, and higher rates of delusional distress. Finally, of the global emotion dysregulation subscales, limited access to emotion regulation strategies was mostly strongly related to distress for both delusional ideations and clinical delusions. In sum, these findings demonstrate that global emotion regulation is significantly impaired in schizophrenia-spectrum disorders and may contribute to delusional distress.

Delusions are comprised of multiple dimensions that shape their severity. Of these, distress is associated with persistence of psychotic experiences,^9^ suggesting it as a critical target for prevention and treatment. While we hypothesized distress to be related to global emotion dysregulation given its association with affective experiences,^24^ we were somewhat surprised to see that global emotion dysregulation was *un*related to delusional conviction on both scales. Yet, this finding is in line with growing literature revealing both cognitive and affective contributions to delusions.^25^ As is being increasingly suggested, delusional conviction may be most tightly linked with cognitive processes, such as reasoning biases and belief inflexibility.^25^ Delusional conviction is also often the stated target in psychological treatments for delusions.^6,26^ This may be most relevant for treatments, such as SlowMo,^6^ that are specifically targeting reasoning biases (eg, jumping to conclusions, belief inflexibility), whereas more affectively-focused interventions (outlined below) could be more beneficial for managing distress.

Within the broader construct of global emotion dysregulation, we found that limited access to emotion regulation strategies was most strongly related to delusional distress. The specific items on this subscale (items 5, 6, 12, 14, and 16) reflect the concept of emotion regulation self-efficacy—an individual’s belief in their ability to manage and change their emotional states.^27,28^ Emotion regulation self-efficacy has been found to be a precursor to effective emotion regulation.^27^ Supporting this, longitudinal research has shown that lower emotion regulation self-efficacy predicts increased depressive and anxiety symptoms over time, even when controlling for general distress.^29^ Similarly, reduced self-efficacy has been linked to greater depressive symptoms following distressing events, such as breaking up with a significant other.^30^

Emotion regulation self-efficacy is strongly associated with decreased psychological functioning, including greater depressive symptoms,^31^ perceived stress^32^ and borderline personality disorder symptoms.^33^ Given this prior research, it is plausible that people who lack confidence in their ability to regulate their emotions—particularly in response to delusions or delusional thinking—may experience heightened distress related to those beliefs. Given this, as well as the link between negative affect and delusion severity,^34^ we were interested to find that limited access to strategies remained a significant predictor of delusional distress in schizophrenia-spectrum disorders, even when controlling for self-reported worry and depression. This suggests that increasing self-efficacy around management of delusional distress specifically, may be a helpful approach.

The social cognitive theory of emotion regulation, which includes emotion regulation self-efficacy, further suggests that successful emotion regulation depends on having clearly defined goals (eg, improving one’s mood) and strategies and behaviors to implement in order to achieve those goals.^27,28^ Items 5 (“When I am upset, I feel like I will remain that way for a long time”) and 12 (“When I am upset, I believe that there is nothing I can do to make myself feel better”) exemplify deficits in these components. These items inversely capture the critical features of successful emotion regulation: belief in one’s ability to implement strategies and achieve emotional change. In a subclinical community sample, lower reappraisal self-efficacy was correlated with more distress associated with psychotic-like experiences^,15^ suggesting its relevance to the schizophrenia-spectrum. In schizophrenia-spectrum disorders, stronger beliefs about the uncontrollability of emotions were linked to greater use of expression suppression, less use of cognitive reappraisal, greater positive symptom severity, and greater severity of depression.^35^*”* Thus, emotion regulation self-efficacy may be a defining factor that exacerbates delusional distress by undermining the individual’s confidence in their ability to manage the emotional impact of their delusions.

In addition, while only found in our schizophrenia-spectrum participants, delusional distress was associated with greater impulse control difficulties, or the feeling that a person cannot be in control of their behavior when they are experiencing negative affect. Previous literature has shown that impulse control difficulties were positively associated with persecutory ideations in subclinical populations.^17^ Therapeutic interventions like SlowMo,^6^ an empirically supported treatment for paranoia, may be helpful in targeting these more behavioral aspects of emotion regulation (like impulse control) that are heavily influenced by negative affect.

Notably, studies have already begun testing the ways global emotion dysregulation can be improved in psychotic disorders. Meta-analysis has shown some promising interventions to help with negative affect and distress in psychotic symptoms, such as acceptance and commitment therapy and compassion-focused therapy.^36^ Both therapies are designed to ease distress and negative emotions associated with psychotic experiences. In addition, Dialectal Behavior Therapy (DBT), which targets emotion regulation as a core skill, has been modified for psychosis and has been found to decrease positive symptom severity and global emotion dysregulation (as measured by the DERS).^37^ In this study, the emotion regulation targets were increased emotional awareness, emotional acceptance, cognitive regulation through reappraisal of whether emotion is driving thought processes, and regulation of physiological responses and impulsive behaviors.^37^ Over the course of 8 weeks, people with schizophrenia-spectrum disorders that underwent a DBT group therapy focused on emotional awareness, understanding and acceptance, and adaptive responses to emotions (through teaching mindfulness, distress tolerance, and emotion regulation skills) reported increased confidence to change persistent negative emotions, and experienced reduced psychological distress.^38^ Future clinical interventions may incorporate emotion regulation self-efficacy as a module in therapy to target delusional distress specifically. Finally, distress is tightly linked with increases in stress,^39^ and recent meta-analyses have found that stress-focused interventions (eg, mindfulness-based stress reduction) effectively reduce depressive symptoms^40^ and have a small positive effect on reducing positive symptoms.^41^ This suggests that reducing stress may help alleviate distress related to psychotic experiences and associated negative affect. Given that negative affect has been found to a play a role in delusions formation and maintenance, developing more standardized, evidence-based global emotion dysregulation interventions are key to reducing delusional distress.^42,43^

There are limitations to this study. The presented results are cross-sectional in nature, limiting our ability to make inferences about temporal dynamics between global emotion dysregulation and delusional distress. Our measure of clinical delusions (PSYRATS) did not assess the persistence of delusions over time, and future research should investigate the relationship between persistent delusions and distress longitudinally. Future research in longitudinal samples will elucidate the interaction between delusional distress and global emotion dysregulation, further aiding treatment recommendations. Given our findings that limited access to emotion regulation strategies was strongly associated with delusional distress, a measure of baseline emotion regulation self-efficacy, and a baseline measure of specific strategy use could have highlighted a specific strategy that is leading to higher (or lower) rates of delusional distress. Finally, the measures used in this study are self-report measures or interview-based self-reports that rely on one’s ability to internally reflect on and report emotional experiences. Emotional awareness is impaired in schizophrenia-spectrum disorders,^3^ and we have found this impairment relates to positive symptom severity^44^ possibly impacting the reliability of these reports.

Another limitation to this study was that childhood trauma and other forms of trauma were not controlled for. It has been found that there is a higher prevalence of childhood trauma in people diagnosed with a psychotic disorder.^45^ Prior literature has shown that childhood trauma is associated with increased severity, frequency, and distress associated with psychotic symptoms.^46^ People who have experienced childhood trauma have also been found to have greater difficulties in regulating their emotions,^47^ and emotion regulation difficulties have been found to partially meditate the relationship between childhood trauma and psychotic symptom distress in subclinical populations.^46^ Given childhood trauma’s link to both psychotic symptoms and emotion regulation and dysregulation, future studies should investigate the relationship between childhood trauma and global emotion dysregulation as it relates to delusional distress. This line of research may be useful for individuals who are at clinical high risk for psychosis, as interventions (specifically CBTp) may be able to target global emotion dysregulation in the context of trauma exposure.

Taken together, this study contributes to the existing literature surrounding global emotion dysregulation and psychosis, by demonstrating across 2 different assessments of delusional thinking that global emotion dysregulation, particularly limited access to strategies, is related to delusional distress. This study provides evidence that global emotion dysregulation is a potential mechanism contributing to delusional distress and is a clinical treatment target for individuals with psychosis. Understanding the specific relationships between affect and positive symptoms are critical to elucidating how delusions are formed and maintained.

## Supplementary Material

sgaf010_suppl_Supplementary_Table_S1
