# Supplementary material for: Delusional Distress is Associated With Emotion Dysregulation in Schizophrenia-Spectrum Disorders
Source: Schizophr Bull Open. 2025 May 23;6(1):sgaf010. doi: 10.1093/schizbullopen/sgaf010 (PMC12216900; doi:10.1093/schizbullopen/sgaf010)

**Table S1**: Demographic and Outcome Variable differences between SZ/SZA and DD/SZF/BPD

| Variable | SZ/SZA, n=81 | DD/SZF/BPD, n=19 | Statistic |
| --- | --- | --- | --- |
| Age, M(SD) | 29.9(9.4) | 29.9(10.9) | t=.009, p=.99 |
| Race, White/Black/Other | 36/33/12 | 12/6/1 | x^2^=8.64, p=.19 |
| Gender, M/F/Other | 56/23/2 | 12/6/1 | x^2^=1.57, p=.67 |
| Personal Ed, M(SD) | 13.5(2.5) | 14.7(1.9) | t=2.01, p=.05* |
| Parental Ed, M(SD) | 14.7(2.7) | 15.1(2.3) | t=.54, p=.59 |
| PDI-21 Total, M(SD) | 87.0(63.2) | 51.4(36.7) | t=-1.85, p=.07 |
| DERS-16 Total, M(SD) | 40.9(17.5) | 36.9(14.3) | t=-.93, p=.35 |
| PSYRATS Total, M(SD) | 13.1(6.01) | 11.4(6.4) | t=-1.02, p=.31 |
| PDI-21 Distress, M(SD) | 2.3(1.3) | 2.02(1.04) | t=. -74, p=.46 |
| PSYRATS Distress, M(SD) | 4.2(3.02) | 3.4(2.9) | t=. -97 p=.33 |
| Tobacco Use, Y/N | 39/42 | 7/12 | x^2^=.24, p=.89 |

Notes: SZ, schizophrenia; SZA, schizoaffective; DD, delusional disorder; SZF, schizophreniform; BPD, brief psychotic disorder; Y, yes, N, no

**Results**

*Global emotion dysregulation, delusion severity, and negative affect*

To assess negative affect and its possible impact on the relationship between delusional ideation and global emotion dysregulation in both people with psychosis and our non-clinical comparison group, participants completed the Beck Depression Inventory (BDI) and the Penn State Worry Questionnaire (PSWQ) (our closest measure of anxiety in both groups was the PSWQ). Multiple linear regressions revealed that even when controlling for negative affect, global emotion dysregulation total scores still significantly predicted total delusional severity on the PDI-21 (F (6, 99) = 19.89, p = .0001, R^2^ = .52). Global emotion dysregulation and PSYRATS total scores were still positively associated when controlling for negative affect, but the relationship was slightly attenuated and no longer significant (F (5, 93) = 7.05, p = .08, R^2^ = .24).

*Global emotion dysregulation, delusional distress, and negative affect*

Because of the attenuated relationship between PSYRATS total scores, depression, and worry, another backward stepwise regression was conducted to see what aspects of global emotion dysregulation would continue to be strongly related to delusional distress for clinical delusions. We continued to find that even when controlling for worry and depression, limited access to emotion regulation strategies (F (4, 94) = 11.25, p = .002, R^2^ = .3) and impulse control difficulties (F (4, 94) = 11.25, p = .01, R^2^ = .3) were still the aspects of global emotion dysregulation that was related to delusional distress above and beyond worry or depressions on the PSYRATS.

**Figure S1**: Intercorrelations Between Variables for Schizophrenia-Spectrum Participants


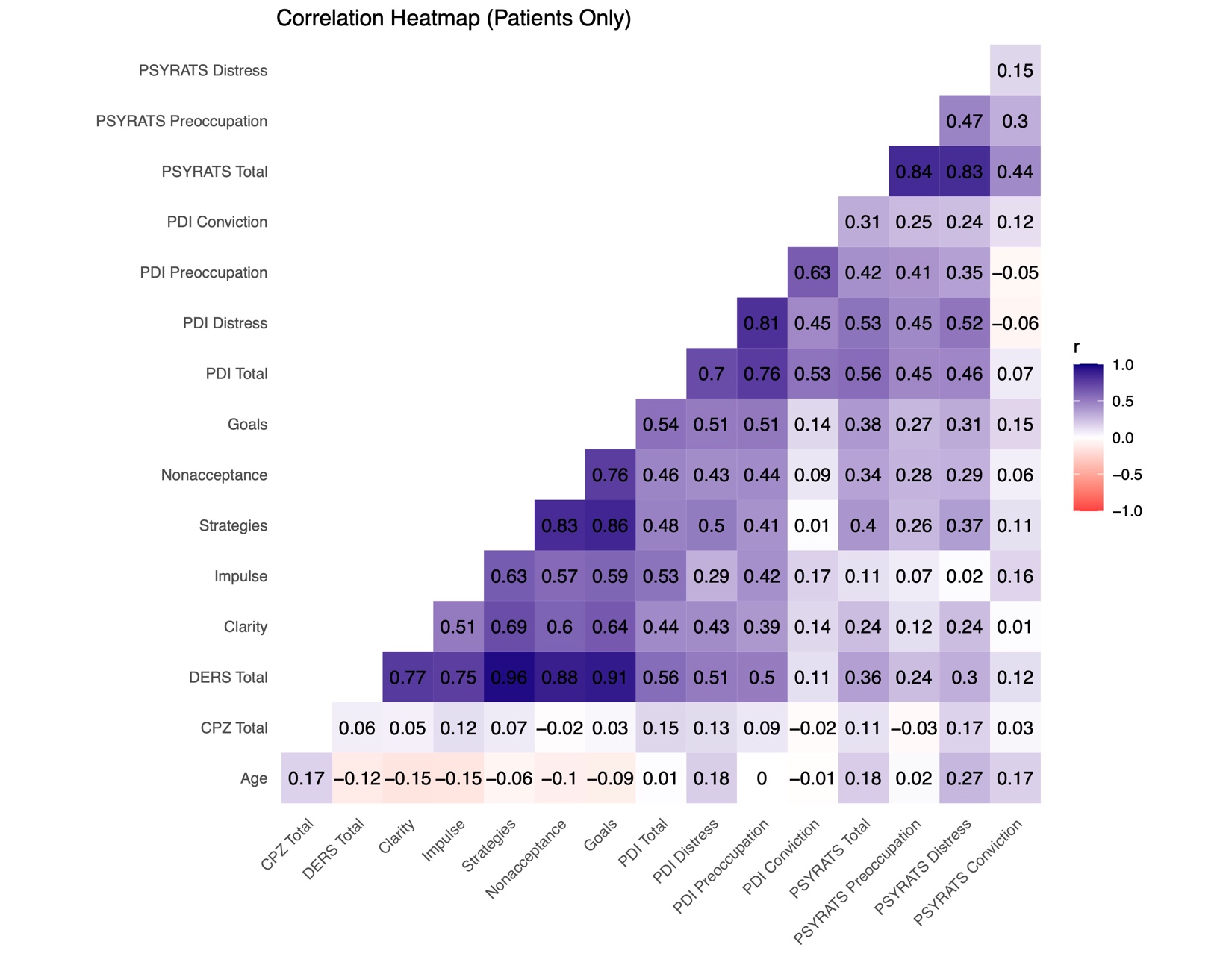


**Figure S2**: Intercorrelations Between Variables for Controls


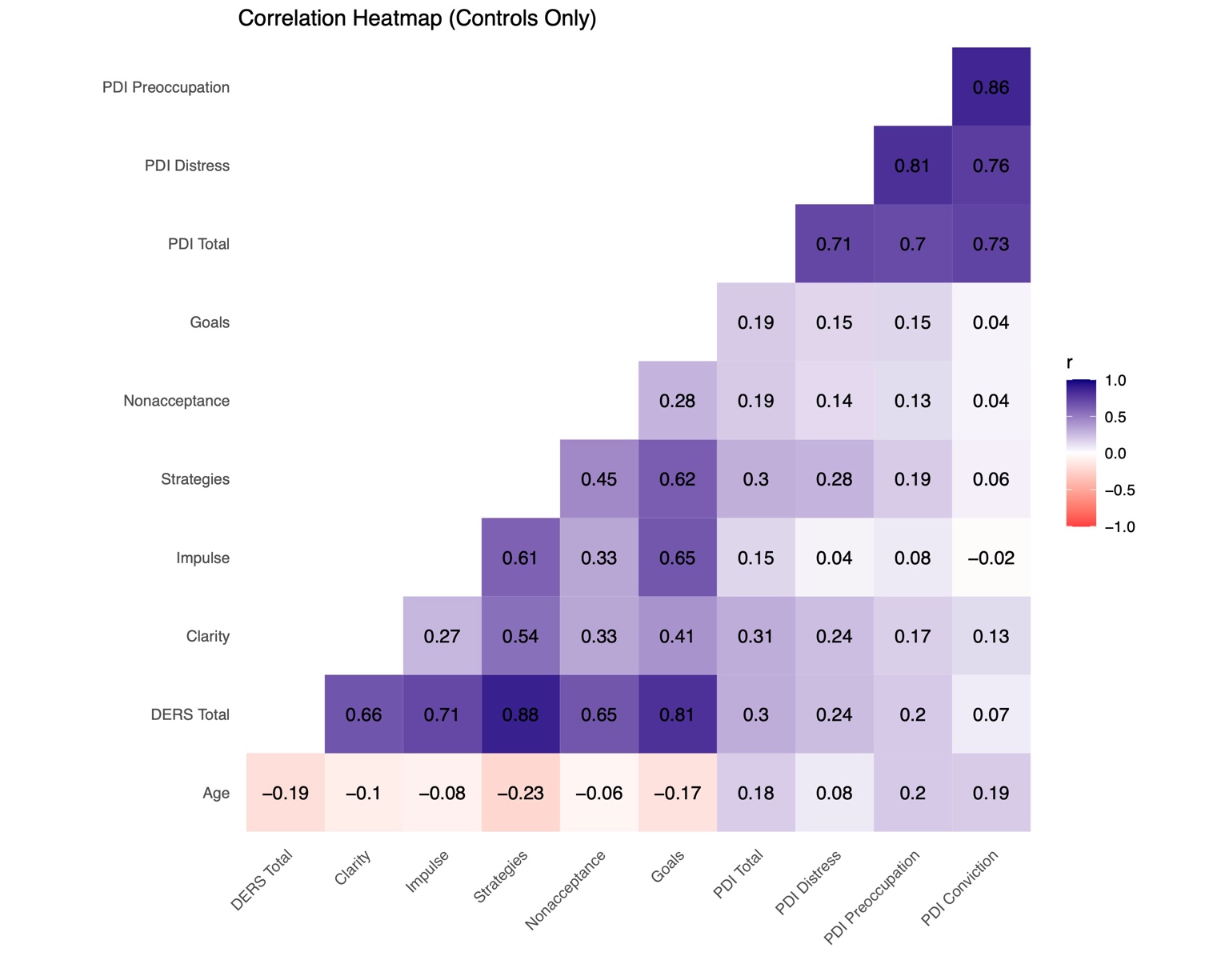

Supplement: sgaf010_suppl_Supplementary_Table_S1 [file sgaf010_suppl_supplementary_table_s1.docx]
